# Psychological Dimensions Relevant to Motivation and Pleasure in Schizophrenia

**DOI:** 10.3389/fnbeh.2022.827260

**Published:** 2022-03-23

**Authors:** Samantha V. Abram, Lauren P. Weittenhiller, Claire E. Bertrand, John R. McQuaid, Daniel H. Mathalon, Judith M. Ford, Susanna L. Fryer

**Affiliations:** ^1^Mental Health Service, Veterans Affairs San Francisco Healthcare System, San Francisco, CA, United States; ^2^Department of Psychiatry and Behavioral Sciences, University of California, San Francisco, San Francisco, CA, United States; ^3^Department of Psychology, University of California, Berkeley, Berkeley, CA, United States

**Keywords:** optimism, cognitive behavioral theory, reappraisal, mindfulness, rumination, social reward sensitivity

## Abstract

Motivation and pleasure deficits are common in schizophrenia, strongly linked with poorer functioning, and may reflect underlying alterations in brain functions governing reward processing and goal pursuit. While there is extensive research examining cognitive and reward mechanisms related to these deficits in schizophrenia, less attention has been paid to psychological characteristics that contribute to resilience against, or risk for, motivation and pleasure impairment. For example, psychological tendencies involving positive future expectancies (e.g., optimism) and effective affect management (e.g., reappraisal, mindfulness) are associated with aspects of reward anticipation and evaluation that optimally guide goal-directed behavior. Conversely, maladaptive thinking patterns (e.g., defeatist performance beliefs, asocial beliefs) and tendencies that amplify negative cognitions (e.g., rumination), may divert cognitive resources away from goal pursuit or reduce willingness to exert effort. Additionally, aspects of sociality, including the propensity to experience social connection as positive reinforcement may be particularly relevant for pursuing social goals. In the current review, we discuss the roles of several psychological characteristics with respect to motivation and pleasure in schizophrenia. We argue that individual variation in these psychological dimensions is relevant to the study of motivation and reward processing in schizophrenia, including interactions between these psychological dimensions and more well-characterized cognitive and reward processing contributors to motivation. We close by emphasizing the value of considering a broad set of modulating factors when studying motivation and pleasure functions in schizophrenia.

## Introduction

Motivation and pleasure deficits are core negative symptoms of schizophrenia and are strongly linked to poor functional outcomes (Fervaha et al., 2014). While considerable research efforts have studied the cognitive and reward processing mechanisms that underlie motivation and pleasure deficits in schizophrenia (Strauss and Gold, 2012; Kring and Barch, 2014; Gold et al., 2015; Najas-Garcia et al., 2018), far less attention has been paid to the role of individual psychological characteristics that contribute to resilience against, or risk for, these impairments. And yet, it is the complex intersection of environmental, psychological, cognitive, and reward processes that both cause and maintain reduced motivation and pleasure in schizophrenia (Strauss et al., 2016). The current review highlights several psychological characteristics that relate to motivation and pleasure dysfunction in schizophrenia and other psychotic disorders, with the goal of broadening the study of amotivation, as a complement to ongoing progress discerning cognitive and reward processing contributions.

Negative symptoms are a cluster of deficits reflecting a loss of typical functioning. These symptoms can be parsimoniously described by two subdomains, one capturing diminished motivation and pleasure (avolition, anhedonia, asociality), and the other capturing diminished expression or communication (blunted affect, alogia) (Blanchard and Cohen, 2006; Blanchard et al., 2011). Relative to expressive deficits, motivation and pleasure deficits are linked to worse outcomes in schizophrenia, including poorer occupational attainment (Llerena et al., 2018) and quality of life (Savill et al., 2016). Motivation and pleasure deficits also better predict real-life functioning and quality of life among individuals at clinical high-risk for psychosis (CHR) (Glenthøj et al., 2020; Üçok et al., 2021). Despite their profound functional impact, there are no established treatments for motivation and pleasure deficits in schizophrenia (Kirkpatrick et al., 2006; Galderisi et al., 2018). To address these treatment gaps, researchers are actively studying the behavioral and neurobiological processes that lead to amotivation and anhedonia in an effort to drive novel therapeutic development. These research efforts focus on disentangling the multifaceted nature of amotivation and anhedonia, to discern how poor reward pursuit may arise from failures at one or more steps in a dynamic sequence (Gold et al., 2008; Barch and Dowd, 2010; Kring and Barch, 2014).

But how do these aspects of brain and behavioral functioning, elaborated by recent research, translate to our understanding of an individual’s presentation in the clinic? That is, how can we refine our definitions of amotivation and anhedonia in terms of clinically relevant psychological processes (Bentall et al., 2010) to capitalize on our improved understanding of the underlying neurobiology? Standard mental health care does not typically make use of the kinds of behavioral or neurobiological assessments used in the laboratory; rather, when treating a patient, clinicians most often rely on retrospective interview techniques to uncover underlying difficulties in experienced pleasure or goal-directed pursuits. For example, Beck conceptualized schizophrenia by adapting his cognitive model of depression, in which he proposed that belief patterns about the self, the world, and the future (termed the cognitive triad) impact a person’s interpretations of events and shape their behavioral responses (Beck et al., 2009, 2019). Negative beliefs about social abilities and resource availability, as well as low expectations for success and pleasure, contribute to and maintain negative symptoms in schizophrenia (Beck and Rector, 2005; Rector et al., 2005). Kingdon and Turkington (2005) further suggest that coping abilities are pivotal to negative symptoms, as negative self-beliefs about performance efficacy and stress management abilities drive amotivation (Rector et al., 2005). These theoretical psychological frameworks are central to treating negative symptoms in schizophrenia using evidence-based behavioral interventions, such as cognitive-behavioral therapy (CBT) for psychosis (Elis et al., 2013). “Third wave” CBT approaches that promote mindfulness, compassion, and self-acceptance (e.g., mindfulness-based cognitive therapy, acceptance and commitment therapy), also show promise for alleviating negative symptoms (Khoury et al., 2013; Jansen et al., 2020). Taken together, psychological beliefs and attitudes are relevant to understanding and treating motivation and pleasure deficits in schizophrenia.

In this review we cover research that relates psychological characteristics with facets of motivation and pleasure. We aim to link laboratory studies of amotivation and anhedonia in schizophrenia with clinically relevant psychological constructs that are typically less considered in clinical neuroscience studies. We focus on approach motivation and positive reinforcement, and therefore do not include studies of avoidance and punishment (though we acknowledge the relevance of negative reinforcement and threat sensitivity to the negative symptoms of schizophrenia). When relevant, we include findings from the depression literature, because amotivation and anhedonia are transdiagnostic features also prevalent in depression (Barch et al., 2016), and because people with schizophrenia experience high rates of concurrent depressive symptoms that impact life satisfaction and motivation (Conley et al., 2007). We first briefly summarize key facets of models of motivation and pleasure deficits in schizophrenia. Next, we discuss dispositional characteristics that relate to psychological beliefs (e.g., optimism/pessimism, defeatist performance beliefs, asocial beliefs), as well as strategies for managing beliefs (e.g., cognitive reappraisal, mindfulness, rumination). We then cover interpersonal psychological tendencies that impact motivation and pleasure in a social context. Finally, we close with recommendations for integrating the study of psychological factors into experimental clinical research on schizophrenia.

## Modeling Motivation and Pleasure Deficits in Schizophrenia: Contributions of Cognitive and Reward Processes

Heuristic models of motivation describe the fundamental and interconnected processes involved in translating hedonic experience into future goal pursuits (Gold et al., 2008; Barch and Dowd, 2010; Kring and Elis, 2013; Kring and Barch, 2014).

### Hedonics

Hedonics refers to in-the-moment pleasure experienced from rewards. Historically, amotivation theories assumed that people with schizophrenia failed to pursue goal-directed activities due to anhedonia, an inability to experience pleasure (Rado, 1956). Empirical evidence over the past two decades yields a different and more nuanced account (Strauss et al., 2014, 2016). While findings from clinical interviews (Kirkpatrick et al., 2011; Strauss and Gold, 2012; Strauss et al., 2012a) and retrospective self-report questionnaires (meta-analysis by Visser et al., 2020) indicate that people with schizophrenia experience less consummatory pleasure, laboratory-based results often contradict this pattern (review by Kring and Moran, 2008). When exposed to putatively pleasant stimuli during laboratory-based assessments, people with and without schizophrenia report comparable levels of positive affect (Cohen and Minor, 2010; Llerena et al., 2012). Similar to laboratory-based studies, people with schizophrenia report equivalent positive affect in daily life when providing in-the-moment responses (or even after a several hour delay; Horan et al., 2006), despite engaging in fewer pleasurable activities relative to controls (Gard et al., 2007; Oorschot et al., 2013; Edwards et al., 2018). This suggests that important distinctions emerge based on whether anhedonia is assessed with momentary assessments of current behavior versus retrospective assessments of past behavior.

More recently, Strauss et al. (2016) argued that the theoretical framework for studying anhedonia in schizophrenia is inadequate. They proposed that the relative balance of positive and negative emotion at a given time point is critical for initiating approach behavior (Larsen et al., 2001). Individuals in the general population exhibit a positivity offset, i.e., they experience more positive than negative emotion to stimuli with lower affective intensity (i.e., more neutral) (Ito and Cacioppo, 2005). This balance is relevant to goal-directed behavior, as routine everyday activities are often more affectively neutral than salient, infrequent, or novel reward opportunities (though everyday goals can be important for attaining longer-term rewards). People with schizophrenia show a diminished positivity offset in situations where the motivational significance is weaker, and this diminished positivity offset correlated with greater self-reported and clinician-rated anhedonia (Strauss et al., 2017). Thus, a reduced positivity offset could help explain why schizophrenia is associated with less reward approach behavior, particularly in response to more neutral affective stimuli. In addition to a reduced positivity offset, other features of positive affect processing have been implicated in schizophrenia, including reductions in sustaining positive emotion (Strauss et al., 2020), and more co-activation of positive and negative emotion to pleasant stimuli than controls, resulting in a lower net hedonic value (Strauss et al., 2017, 2020). These data suggest that increased persistence of negative emotion, and decreased persistence of positive emotion, are both relevant to the clinical observation of anhedonia.

### Reward Anticipation

Deficits in reward anticipation and reward learning have been put forth as key mechanisms for understanding motivation deficits in schizophrenia (Strauss et al., 2014; Barch et al., 2016; Waltz and Gold, 2016). Reward anticipation depends, in part, on prospection (the ability to imagine future experiences; Gilbert and Wilson, 2007) and episodic memory (the encoding and subsequent recall of past experiences) (Addis et al., 2007; Schacter and Addis, 2007). Prospection can induce in-the-moment positive emotion that helps an individual to predict their hedonic reaction to future events (i.e., affective anticipatory forecasting) (Frost and Strauss, 2016). People with schizophrenia have well-described episodic memory impairments (reviewed by Leavitt and Goldberg, 2009), and generate less clear and detailed future prospections, with less self-referential detail (D’Argembeau et al., 2008; Raffard et al., 2013; Painter and Kring, 2016). Moreover, they report less anticipated enjoyment from future experiences, which may undermine motivation (Kring and Barch, 2014). Meta-analytic evidence indicates a small-to-moderate reduction in reward anticipation in schizophrenia relative to unaffected comparison participants (Hallford and Sharma, 2019); further, longer illness duration and worse cognitive functioning predict greater deficits in reward anticipation. Schizophrenia is also associated with deficits in the maintenance of stimulus and reward relationships over time (Herbener, 2009), and poor maintenance of reward information may relate to anticipatory pleasure deficits (Wang et al., 2020).

### Reward Learning

The ability to adapt behavior based on experience is critical for reward attainment. Reinforcement learning has two components: (i) learning associations that lead to reward (via positive reinforcement), and (ii) learning associations to avoid loss (via punishment) (Moran et al., 2019). Essentially, reward learning is the process by which an individual adjusts their future expectations and actions when faced with an outcome that differs from what was expected (Sutton and Barto, 2018). Mismatches between expected and obtained outcomes yield reward prediction errors, which are used to update value estimations and drive reward learning (Niv and Schoenbaum, 2008; Glimcher, 2011). Better-than-expected outcomes (i.e., positive prediction errors) facilitate goal pursuit, whereas worse-than-expected outcomes reduce goal pursuit, in a context-dependent manner.

Schizophrenia is associated with deficits in behavioral updating following feedback (Strauss et al., 2014), and in particular, learning from positive reinforcement (Waltz et al., 2007; Cicero et al., 2014; Dowd et al., 2016; Barch et al., 2017; Hartmann-Riemer et al., 2017). Reward information retention also degrades more rapidly as a function of negative symptom severity (Culbreth et al., 2021). Furthermore, working memory deficits are related to reward learning deficits in schizophrenia (Collins et al., 2014, 2017; Barch et al., 2017), suggesting that the ability to maintain environmental contingencies supports reinforcement learning. Thus, cognitive functions, like episodic and working memory, are central to understanding reward learning deficits in schizophrenia.

### Value and Effort Computations

Behavioral and neuroimaging evidence suggest that amotivation in schizophrenia is influenced by difficulties representing and flexibly updating reward value in the context of learning (reviewed by Waltz and Gold, 2016). Importantly, motivated behavior is not only driven by representation of reward value, but also by computations about the benefits relative to the costs of the required work (Treadway et al., 2009). Alterations in cost-benefit calculations are thought to be related to motivation deficits in schizophrenia (Waltz and Gold, 2016). Accordingly, the willingness to exert effort has become an important focus of schizophrenia research (Culbreth et al., 2018). The representation of effort-cost computations can be modeled in the laboratory with simple motor tasks, such as the Effort Expenditure for Rewards (EEfRT) task (Treadway et al., 2009), which asks participants to choose between an easy (i.e., fewer button presses, resulting in smaller rewards) or hard (i.e., more button presses, resulting in larger rewards) option. Several studies report that people with schizophrenia are less likely to select the hard option (Fervaha et al., 2013; Barch et al., 2014; Huang et al., 2016), and that reduced effort exertion correlates with amotivation (Barch et al., 2014; Wolf et al., 2014; Moran et al., 2017). Similar findings of reduced effort in schizophrenia have been observed using tasks of cognitive effort (Culbreth et al., 2016b; Chang et al., 2020).

## Psychological Dimensions Relevant to Amotivation and Anhedonia

Despite being key drivers of behavior, variation in personality or other psychological dimensions are not commonly studied in the cognitive sciences (Carver and Scheier, 2014). These psychological features may explain unique variance in motivation and pleasure deficits or interact with other features related to motivation and pleasure that are well-characterized in schizophrenia, as described above. Further, such cognitive-psychological interactions are rarely examined in the schizophrenia literature.

### Optimism

Optimism is the tendency to expect positive outcomes in the future (Sharot, 2011), reflecting a confident, context-independent disposition toward life (Carver and Connor-Smith, 2010). Higher levels of optimism are associated with greater well-being across cultures and geographic regions (Gallagher et al., 2013), and with greater life satisfaction in people with schizophrenia (Seo and Lim, 2019).

Although more commonly studied in personality research than cognitive science, optimism can be construed as a cognitive construct, as it relies on future expectancies (Carver and Scheier, 2014). Consequently, optimism is relevant to the kinds of expectancy-value calculations involved in goal-directed behaviors (Carver and Connor-Smith, 2010). For instance, optimistic individuals are more confident about their likelihood of success and are willing to exert greater effort to attain a desired goal, as compared to pessimistic individuals (Nes et al., 2005; Carver and Connor-Smith, 2010). Trait-level optimism is also linked with reward expectations and learning. More optimistic individuals over-estimate the likelihood of reward from uncertain targets during Pavlovian conditioning (Stankevicius et al., 2014), consistent with basic science findings in which “optimistic” rats are more motivated than “pessimistic” rats to work for rewards (Rygula et al., 2015). And while purely rational agents should adjust their expectations when presented with disconfirming evidence (Sutton and Barto, 2018), humans update their expectations more in response to positive than negative feedback, consistent with a normative positivity/optimism bias (Sharot et al., 2011; Garrett and Sharot, 2017; Lefebvre et al., 2017; Marks and Baines, 2017). Conversely, the optimism bias is often absent or even reversed in people with depression (Strunk et al., 2006; Strunk and Adler, 2009; Garrett et al., 2014; Korn et al., 2014).

Few studies have examined optimism bias in schizophrenia, leaving open questions about whether the expected positivity bias is intact. In a sample of people with schizophrenia and unaffected comparison participants, Prentice et al. (2005) measured optimism bias as the extent to which a given participant rated their likelihood of experiencing adverse events as lower than others. The authors found that, while both groups showed unrealistic optimism in general, comparison participants endorsed higher optimism for controllable events (e.g., losing money while gambling). This finding is consistent with the idea that individuals with schizophrenia experience a reduced internal locus of control (i.e., less perceived control over outcomes as a result of personal ability/agency), as previously suggested by Fischer et al. (2015).

Optimism levels could moderate motivation through relationships with reward anticipation and reward learning. Because mental imagery is needed to simulate future reward outcomes, failures to generate rich positive future imagery may impact current and predicted pleasure in schizophrenia (Frost and Strauss, 2016; Painter and Kring, 2016; Yang et al., 2018). In a non-psychiatric community sample, dispositional optimism correlated with more vivid lab-assessed imagery of positive future events (Blackwell et al., 2013). While imagery has not been studied in connection with optimism in schizophrenia, the ability to simulate positive future imagery is impaired in dysphoric (Holmes et al., 2008) and depressed (Morina et al., 2011) individuals. The vividness of positive prospective imagery correlates with dispositional optimism in depression, suggesting meaningful heterogeneity in prospection as a function of trait optimism (Ji et al., 2017). Relative to unaffected comparison participants, people with schizophrenia generate less detailed and specific future imagery (meta-analysis by Hallford et al., 2018). Episodic memory deficits may contribute to poorer prospection (Frost and Strauss, 2016), based on evidence that people with schizophrenia are less likely to draw from past experiences during prospection (Painter and Kring, 2016). In addition to the role of memory, we (and others; Chen et al., 2016) speculate that individual differences in dispositional optimism may correlate with positive future imagery abilities in schizophrenia; that is, greater negative self-beliefs and reduced optimism may relate to deficits in positive imagery generation (Raffard et al., 2013; Chen et al., 2016).

Optimism may also influence how reward expectations are updated. Non-clinical participants with higher trait optimism show less encoding of undesirable information during functional magnetic resonance imaging (fMRI), as reflected by reduced fMRI activation in prefrontal cortex relative to less optimistic individuals (Sharot et al., 2011). Non-clinical participants also incorporate better-than-expected outcomes at a higher rate than worse-than-expected outcomes in a reward learning task, and more optimistic responders had a higher learning rate for positive relative to negative prediction errors, with pessimistic responders showing the opposite pattern (Lefebvre et al., 2017). Several studies report a selective deficit in learning from positive outcomes among people with schizophrenia, with learning from loss relatively spared (Waltz et al., 2007; Strauss et al., 2011; Gold et al., 2012; Hartmann-Riemer et al., 2017; though see Barch et al., 2017). An intriguing question is whether variation along the optimism–pessimism dimension relates to reward learning impairments in schizophrenia. For example, it is unknown whether higher levels of pessimism relate to overestimation of negative prediction errors in schizophrenia (and whether corresponding neural circuitry could explain such a relationship).

### Negative Thinking Tendencies About the Self, Others, and the Future

Beck’s cognitive model of depression (Beck, 1967) postulates how early adverse events (e.g., childhood trauma, genetic or other biological predispositions) can contribute to the development of maladaptive cognitive schemas (e.g., “I am unlovable,” “things will never improve.”), negative emotions (e.g., sadness, hopelessness), and behavioral responses (e.g., withdrawal). Dysfunctional beliefs can bias attention and memory processes, thereby creating a negative feedback loop that perpetuates negative affect and can contribute to psychopathology. Beck later adapted his original cognitive model of depression to conceptualize schizophrenia, including negative symptoms and poor functional outcomes (Rector et al., 2005; Beck et al., 2009). Beck’s conceptualization of schizophrenia describes how biological vulnerabilities and/or discouraging experiences (e.g., social rejection, poor school performance) in combination with illness features (e.g., neurocognitive deficits) can develop and maintain low expectations for the future that foster amotivation (Beck et al., 2018). Within Beck’s model, the cognitive triad parses dysfunctional beliefs into thoughts about (i) the self, (ii) others, and (iii) the future (Clark et al., 1999); each of which has been found to correlate with amotivation in the general population (Pillny et al., 2018). Conversely, aspects of psychological well-being, such as experiences of autonomy, personal growth, and positive relations with others, correlate with less amotivation in people with schizophrenia (Strauss et al., 2012b).

#### Defeatist Performance Beliefs

Defeatist performance beliefs are one type of negative self-beliefs, and are characterized by overgeneralized, negative conclusions about one’s ability to execute goal-directed behaviors (e.g., “why even try, I always fail”). While historically conceptualized in the context of depression, research shows elevated defeatist performance beliefs in people with schizophrenia (Grant and Beck, 2009; Bentall et al., 2010; Horan et al., 2010), people with schizotypy (Luther et al., 2016), and CHR participants (Clay et al., 2021). A meta-analysis found that defeatist performance beliefs predict a small, but significant proportion of variance in negative symptoms (Campellone et al., 2016b), with many (although not all, McGovern et al., 2020) studies directly linking defeatist performance beliefs with motivation deficits in schizophrenia (Horan et al., 2010; Couture et al., 2011; Green et al., 2012; Ventura et al., 2014; Granholm et al., 2016; Lee and Yu, 2021). Further, defeatist performance beliefs may mediate both the relationship between neurocognitive deficits and negative symptoms among people with schizophrenia (Horan et al., 2010; Quinlan et al., 2014), and the relationship between role functioning and negative symptoms among CHR participants (Devoe et al., 2021).

Defeatist performance beliefs are associated with reduced willingness to expend effort in schizophrenia. For example, defeatist performance beliefs moderate the relationship between effort expenditure and clinical ratings of amotivation and anhedonia, in that individuals with the highest levels of defeatist performance beliefs and motivation and pleasure deficits put forth the lowest effort (Reddy et al., 2018). That is, negative performance beliefs about one’s own capabilities corresponded with less empirically measured effort. In a study where pupillary responses during a memory task indexed cognitive effort, Granholm et al. (2016) found that schizophrenia participants with severe defeatist performance beliefs did not increase effort. (Although a subsequent study measuring pupillary responses did not detect a direct relationship between cognitive effort and defeatist performance beliefs; McGovern et al., 2020). Defeatist performance beliefs also correlated with positive and negative schizotypy traits in non-clinical college students, with higher defeatist performance beliefs predicting less real-life goal progress, effort expenditure, and pleasure experience (measured from daily surveys; Campellone et al., 2019). Very little research has examined how defeatist performance beliefs relate to physical effort expenditure in schizophrenia, though one study did not observe a relationship between defeatist performance beliefs and willingness to exert force (Décombe et al., 2021).

In contrast to defeatist performance beliefs, self-efficacy reflects a person’s belief in their capacity to successfully execute the behaviors needed to attain a specific goal. Expectations of success positively correspond with behavior initiation and sustained effort (Bandura, 1977). For instance, self-efficacy moderates the relationship between laboratory-based functional capacity measures and self-reported daily functioning. When self-efficacy was high, there was a positive correlation between functional capacity and real-world functioning in schizophrenia participants, that was absent in a low self-efficacy sub-group (Cardenas et al., 2013); thus, self-efficacy may help explain why some individuals are better at translating ability into real-world pursuits than others. Importantly, the connection between self-efficacy and functioning may depend on clinical insight (Kurtz et al., 2013) and negative symptom severity (Pratt et al., 2005; Ventura et al., 2014), highlighting important individual difference factors that relate to self-efficacy and achievement outcomes in schizophrenia.

#### Asocial Beliefs

Fewer studies have examined how asocial beliefs (e.g., “I am better off alone,” “making new friends is not worth the effort”) impact motivation and pleasure in schizophrenia. Asocial beliefs, which fall under negative beliefs about others in Beck’s model, may arise from negative social experiences that contribute to reduced social engagement (Grant and Beck, 2010). Negative views of oneself or others might reduce social motivation, preventing the occurrence of more positive and corrective social experiences for people with psychosis (Rector et al., 2005; Jaya et al., 2017). This notion is supported by evidence that repeated experiences of experimentally induced social exclusion (e.g., during the Cyberball task) correlate with demotivating beliefs and motivational deficits (Pillny and Lincoln, 2020). Asocial beliefs correlate with less social behavior in people with schizophrenia, accounting for more unique variance in social functioning than negative or depressive symptoms (Grant and Beck, 2010). Additionally, asocial beliefs predict community participation among individuals with a psychotic disorder (Thomas et al., 2017), and mediate the effects of social functioning on negative symptoms in CHR participants (Devoe et al., 2021).

#### Low Expectancy Beliefs About the Future

The final component of the cognitive triad is negative views about the future. This component includes low expectancy beliefs about future pleasure (e.g., “I won’t enjoy the activity”), which can be (inversely) conceptualized as anticipatory pleasure beliefs, depending on the measurement scale. These beliefs are similar to dispositional optimism (or rather pessimism) in their future orientation, but low expectancy beliefs are more situationally specific, whereas optimistic/pessimistic beliefs reflect generalized expectations across contexts (Kube et al., 2018). Findings from a recent meta-analysis indicate that people across the schizophrenia spectrum self-report lower levels of anticipatory pleasure beliefs (Visser et al., 2020). Prior research links low expectations of future pleasure with amotivation in schizophrenia (Da Silva et al., 2017). One theory is that anticipatory pleasure beliefs mediate the translation of goal-intentions into goal-directed behavior (Pillny et al., 2020).

The relationship between anticipatory pleasure beliefs and motivation is evident from experimental studies of effort expenditure and reward learning. In a study using the EEfrt task in a university sample, anticipatory pleasure beliefs positively correlated with high-effort option selection when the probability of reward delivery was low (Geaney et al., 2015); that is, individuals who reported greater future pleasure expectancies were more willing to expend effort for larger but less likely rewards, suggesting a path by which pleasure expectancies may mitigate perceived effort costs to drive goal-pursuit. Anticipatory pleasure beliefs were also linked with the decision to pursue high-effort reward options among undergraduate students (Olino et al., 2021), and with greater persistence of effortful responding in individuals without a psychiatric history (DeRosse et al., 2019; though see Zou et al., 2020). Additionally, anticipatory pleasure beliefs relate to reward prediction signaling in non-clinical adults. Cooper et al. (2014) observed a positive relationship between self-reported anticipatory pleasure beliefs and the reward positivity (RewP) event-related potential (ERP) component. More specifically, people who expect more future pleasure appear to have enhanced neural signals related to reward learning and feedback. While the relationship between anticipatory pleasure beliefs with effort expenditure and reward learning in schizophrenia is unknown, this emerging body of work encourages additional study.

### Reappraisal, Mindfulness, and Rumination: Navigating Negative Thought Patterns

Having covered how different psychological beliefs relate to motivated behavior in schizophrenia, we now discuss how individual variation in managing those beliefs may interact with motivation and pleasure processes.

#### Cognitive Reappraisal

Cognitive reappraisal is an emotion regulation strategy that involves mentally altering the meaning of a situation to mitigate its emotional impact (Gross, 1998, 2015). Reappraisal occurs early in the emotion generative process and is relatively more effective than response-focused strategies, like suppression (Gross, 2001). Reappraisal can be studied via experimental induction (i.e., participants are explicitly instructed to reappraise) or with trait measures that assess frequency of reappraisal use in daily life. Greater habitual use of reappraisal correlates with greater psychological well-being (Gross and John, 2003; McRae et al., 2012), and more resilience against depressive symptoms in the context of high stress (Troy et al., 2010).

Similar to findings from the general population, greater use of reappraisal correlates with more positive emotion in schizophrenia (Moran et al., 2018), highlighting a meaningful relationship between emotion regulation and affective experience. However, meta-analytic findings indicate suboptimal emotion regulation in schizophrenia, characterized by overreliance on less effective strategies such as rumination or distraction, along with a reduction in adaptive strategies like reappraisal (Ludwig et al., 2019). Strauss et al. (2016) theorize that deficits in managing negative emotion (e.g., reappraisal) may result in a net hedonic balance of more negative than positive emotion; this net negative balance may contribute to goal-directed and pleasure-seeking deficits, as a higher ratio of positive to negative emotion is thought to support motivational drive (Fredrickson, 2013). This is consistent with the assertion that people with schizophrenia have difficulty pursuing goals, especially in the context of negative emotion (Lawlor et al., 2020). For instance, relative to unaffected comparison participants, people with schizophrenia report greater difficulty pursuing goals while concurrently experiencing or anticipating negative emotions (Lincoln et al., 2015).

Etkin et al. (2015) recently proposed that emotion regulation can be couched within a reinforcement learning framework. From this perspective, reappraisal entails flexibly adjusting one’s emotional response model based on new contextual details, a working model of the environment, and one’s internal state. Decisional control within the reinforcement learning framework comprises both model-free (behavior guided entirely from prediction errors, assuming no prior knowledge) and model-based (behavior guided from rules and dynamic computation of optimal actions based on existing knowledge of that context). “Model-based” calculations have been shown to be deficient in schizophrenia when studied using a reward decision task (Culbreth et al., 2016a), and might help explain why individuals with schizophrenia are less effective at implementing reappraisal using both within-subject (Strauss et al., 2019), and case-control (Visser et al., 2018) experimental designs.

Reappraisal has been linked with reward cues and outcome responses in the general population. Using a reward time-estimation task in non-clinical participants, Kelley et al. (2019) found that individual variation in reappraisal tendencies correlated with more salience to rewarding cues (indexed by the cue-P3 ERP component), but not with ERP components related to reward anticipation or outcome processing; this fits with notions that reappraisal may exert its influence at a relatively early point in the emotion processing stream. In contrast to the positive relationship between self-reported reappraisal strategies and reward cue processing, studies that experimentally induce or manipulate reappraisal report findings consistent with appraisal having attenuating effects on reward outcome processing. For instance, during a gambling task, when undergraduate students were asked to use cognitive reappraisal, they self-rated reduced emotional responses to gains and losses (i.e., gains were less positive and losses less negative, when compared to a control condition) (Yang et al., 2013). Similarly, reappraisal led to blunting of the feedback-related negativity (FRN) and P3 ERP components, which capture early and later-stage outcome evaluation, respectively.

Further, in studies of the late positive potential (LPP) ERP component, the expected reduction in reactivity to unpleasant images observed in controls during reappraisal induction is reduced in schizotypy (Pan et al., 2020), and schizophrenia (at a trend level; Bartolomeo et al., 2020). Participants with schizophrenia also showed deficient pupil dilation during reappraisal relative to controls, suggesting that low cognitive effort is associated with ineffective reappraisal use (reflected by similar LPP amplitudes for the reappraisal and passive viewing conditions; Bartolomeo et al., 2020). Additional research is needed to test whether dispositional reappraisal similarly relates to effort in schizophrenia.

#### Mindfulness and Savoring

Mindfulness reflects attention to the present moment in a non-judgmental, non-reactive manner (Kabat-Zin, 1990; Brown and Ryan, 2003). It can be studied as an inherent individual difference (i.e., trait or disposition) (Baer et al., 2004), or one that is cultivated through practice (Kiken et al., 2015). For the purposes of this review, we focus on dispositional mindfulness. Greater dispositional mindfulness in the general population is associated with increased attention to internal experience, including awareness of emotions and somatic states (Shapiro et al., 2006; Hanley et al., 2017). Dispositional mindfulness positively correlates with health behaviors across psychiatric and non-psychiatric samples (meta-analysis by Sala et al., 2020). Meta-analytic findings also indicate that mindfulness relates more to intrinsic, than extrinsic, motivation (Donald et al., 2020), i.e., mindfulness relates to the pursuit of an activity because of genuine interest and enjoyment versus pursuit to attain an external outcome, like praise or money (Ryan and Deci, 2000).

Mindfulness impacts the awareness, labeling, and experience of emotions (Heppner et al., 2015), and awareness and acceptance of emotion are thought to be central features of emotion regulation (Gratz and Roemer, 2004). Accordingly, mindfulness may influence motivational processes through emotion regulatory mechanisms (Tang et al., 2015). Mindfulness meditation can be construed as a form of affective exposure, as meditators are taught to accept and tolerate negative internal experiences without responding (Baer, 2003; Treanor, 2011). Studies in non-psychiatric samples indicate trait mindfulness relates to greater self-reported mood regulation (Lyvers et al., 2014; Stevenson et al., 2019; Jin et al., 2020), an increased ability to let go of negative thoughts (Frewen et al., 2008), and a reduced frequency of negative automatic thoughts (Frewen et al., 2008). Electrophysiological studies of undergraduate students further exemplify the positive relationship between mindfulness and affective stability (review by Rau and Williams, 2016). For instance, more mindful individuals show attenuated responses to highly arousing unpleasant images (indexed by the LPP) (Brown et al., 2013), and less differentiation between reward and neutral feedback (indexed by the RewP) (Teper and Inzlicht, 2014). Additionally, higher mindful awareness and positive reappraisal predict greater self-efficacy following perceived failures (Hanley et al., 2015). Thus, the ability to mindfully attend to and/or positively regulate emotions may support goal maintenance in the face of unexpected or aversive obstacles. Mindfulness may also help individuals sustain effort in pursuit of a valued distal goal, even when the pursuit itself is not inherently rewarding, or is even aversive, like quitting and maintaining abstinence from cigarette smoking (Heppner et al., 2016).

There is a paucity of basic research on dispositional mindfulness in schizophrenia (Martins et al., 2017), despite the increasing use of mindfulness-based interventions for treating people with psychotic disorders (meta-analysis by Jansen et al., 2020). A foundational study by Tabak et al. (2015) found that people with schizophrenia reported lower levels of self-reported mindfulness relative to unaffected comparison participants (although others did not observe these group differences; López-Del-Hoyo et al., 2019). Less dispositional mindfulness correlates with worse overall symptom severity in people with schizophrenia (Hochheiser et al., 2020), including greater clinician-rated amotivation (Lee and Yu, 2021). Moreover, higher trait mindfulness is associated with self-report of more successful emotion regulation (increased cognitive reappraisal) in schizophrenia (Tabak et al., 2015). The degree to which the interplay between mindfulness and reappraisal enhances goal pursuit in schizophrenia thus warrants additional research.

Conceptually related to mindfulness is the construct of savoring (for the purposes of this review we refer specifically to “savoring the moment” versus savoring through reminiscence or anticipation) (Bryant and Smith, 2015). Savoring is the attention to, appreciation, and enhancement of positive experiences (Bryant and Verhoff, 2007). Although mindfulness and savoring are moderate-to-highly correlated (Beaumont, 2011; Kiken et al., 2017; Cheung and Lau, 2021), savoring is distinct from mindfulness in that it is restricted to positively valenced experiences (in contrast to mindfulness, which encompasses all aspects of current experience, regardless of positive, negative, or neutral valence) (Bryant and Verhoff, 2007). Savoring correlates with more positive emotion, particularly for individuals with high and moderate (but not low) levels of mindfulness (Kiken et al., 2017). Moreover, savoring correlates with more positive emotion during negative events, and lower negative emotions during positive events, which, overall, may help to amplify net positive emotion (Ma et al., 2020). People with schizophrenia report less savoring than unaffected comparison participants, which may impede the up-regulation of positive affect (Cassar et al., 2013). Greater self-report of savoring in people with schizophrenia also corresponds with more positive emotion, less negative emotion, and less clinician-rated amotivation (Moran et al., 2018). Taken together, both mindfulness and savoring relate to affective responsiveness in ways that are relevant to goal-directed behavior.

In contrast to mindfulness, active avoidance of negative internal experiences may undermine goal pursuit in schizophrenia. Experiential avoidance, which is inversely correlated with mindfulness (McCluskey et al., 2020), is the reluctance to have difficult internal experiences like negative thoughts or feelings, resulting in the avoidance or suppression of those negative internal experiences (Hayes et al., 2004). Willingness to experience negative emotions can be necessary for pursuing meaningful activities and attaining goals (Lawlor et al., 2020). People with schizophrenia report greater experiential avoidance than controls (Goldstone et al., 2011; Vorontsova et al., 2013). Whether experiential avoidance directly relates to motivation and pleasure functions in schizophrenia has not been well-studied.

#### Rumination

The process of carefully or repetitively thinking is called rumination, with trait rumination often conceptualized as a mental habit of repetitive self-focused thought (Watkins and Nolen-Hoeksema, 2014). Researchers typically study negative forms of rumination and rumination-associated distress (Nolen-Hoeksema, 1991; Nolen-Hoeksema et al., 2008), which we focus on here, though we recognize growing interest in a broader dimension that includes positive rumination (Li et al., 2017; Yang et al., 2020).

Rumination amplifies a particular mood state. In the case of negative affect, rumination leads to elongated periods of depression, and thus increases risk for developing a depressive disorder (Nolen-Hoeksema et al., 2008). Whitmer and Gotlib (2013) hypothesize that a narrowing of attention in the context of negative affect can lead to a repetitive focus on negative thoughts, thus exacerbating negative mood states. Depressive rumination has been shown to covary with dysfunctional cognitions (Yapan et al., 2020), and interact with dysfunctional cognitions to predict the onset, number, and duration of future depressive episodes (Robinson and Alloy, 2003). In effect, rumination can enhance the harmful impact of negative schemas and cognitions. Unsurprisingly, rumination is inversely related to more adaptative thinking tendencies, like cognitive reappraisal (Beath et al., 2019).

Meta-analytic results indicate that there is an overreliance on rumination in schizophrenia (Ludwig et al., 2019). Moreover, rumination correlates with worse negative symptom severity (Halari et al., 2009), and greater positive symptom severity, such as persecutory ideation and auditory hallucinations (Hartley et al., 2014). More specifically, rumination may impact the balance of positive and negative affective experience, and co-activation of negative emotion may reduce the net hedonic value of stimuli rendering them less rewarding (Strauss et al., 2017). This notion is supported by empirical evidence that rumination induction increases negative affect and decreases positive affect in undergraduate students (McLaughlin et al., 2007). Further, momentary levels of rumination correlate with subsequent increases in negative affect and decreases in positive affect, among individuals with major depressive disorder and/or generalized anxiety disorder (Kircanski et al., 2018).

In addition, rumination may disrupt goal engagement and reward learning processes. For instance, when asked to solve anagrams, ruminators were less likely to skip an unsolvable anagram (van Randenborgh et al., 2010). This suggests that trait rumination corresponds with difficulties disengaging from unrealistic goals. With respect to reward learning, Takano et al. (2019) found that ruminative brooding correlates with worse performance on an emotional reversal learning task; in particular, high-trait ruminators were less able to detect changes in action-outcome contingencies on the probabilistic reinforcement task. In the depression literature, rumination is associated with greater elaboration of losses than gains during a gambling task, pointing to enhanced or sustained processing of negative information (Webb et al., 2017). To date, the impact of rumination on reward learning in schizophrenia has not been thoroughly examined. However, given the literature highlighting reward learning deficits in schizophrenia (Gold et al., 2008; Strauss et al., 2011; Waltz et al., 2011, 2018; Schlagenhauf et al., 2014), this is an area that merits investigation.

### Social Motivation: Sensitivity to Reward and Approach Goal Orientation

Motivation is a process that occurs within the individual and can be shaped by internal psychological characteristics. However, motivation is also influenced by social context (Finkel et al., 2016). Social connection is central to motivation, as sharing goals with others can facilitate goal commitment (Ingledew et al., 2005), and receiving social support enables goal pursuit (Jakubiak and Feeney, 2016). Furthermore, social connection is a motivationally salient reward in and of itself (Baumeister and Leary, 1995). People with schizophrenia share a desire for social connection that is similar to those without the illness; they report similar levels of social interest (Trémeau et al., 2013) and are motivated by the same aspects of social connection (Weittenhiller et al., 2021). However, people with schizophrenia set fewer social goals in their daily lives (Gard et al., 2014) and report spending more time alone (Kasanova et al., 2018). What underpins the mismatch between intention and action? Social motivation theory posits that individual differences in sensitivity to social reward and threat underlie the translation from interest to behavior (Gable, 2006). Thus, social reward sensitivity may help explain social motivation in schizophrenia.

#### Modeling Social Motivation

According to Gable’s (2006) social motivation model, variation in social reward and threat sensitivity influence how people conceptualize their social goals, experience social connection, and define relational success. Social reward sensitivity (i.e., strength of social approach motive) refers to the degree to which positive reinforcers associated with social connection motivate goal-directed behavior (Russell and Mehrabian, 1978). This differs from sensitivity to social threat, which is primarily concerned with avoiding social punishments. We focus on social reward sensitivity in this review, as our emphasis is on approach motivation, and thus sensitivity to threat is outside our purview. Nonetheless, we acknowledge that the combination of diminished social reward sensitivity coupled with heightened social threat sensitivity may be particularly important to the conceptualization of social amotivation (Fulford et al., 2018).

#### Social Reward Sensitivity

Social reward sensitivity is a relatively stable trait that is separable from more generalized incentive sensitivities (e.g., behavioral activation system [BAS]) or attachment styles (Gable and Gosnell, 2013). In line with social motivation theory, individual differences in social reward sensitivity predict how people frame their social goals, such that people who are higher in this trait are more likely to adopt approach-oriented goals (e.g., make a new friend, spend quality time with their spouse) (Gable, 2015). Social reward sensitivity is also theorized to influence how people define success in relationships. Individuals with higher social reward sensitivity have more positive expectancies, leading them to behave in a way that elicits more positive responses from others (Gable, 2006).

Individual differences in social reward sensitivity correlate with interpersonal and intrapersonal outcomes, including social life satisfaction (Gable, 2006), feelings of belongingness (Nikitin and Freund, 2018), and subjective well-being (Nikitin and Freund, 2019). Diminished social reward sensitivity relates to social motivation difficulties (Fulford et al., 2018), which affect approximately half of people with schizophrenia (Bobes et al., 2010). In a study of general reward sensitivity, Reddy et al. (2014) identified a subgroup of people with schizophrenia who had both diminished reward sensitivity and diminished social motivation; this group had particularly poor social functioning, underscoring the negative impact associated with motivational difficulties. People with schizophrenia also show reduced social reward-related caudate nucleus activation during a cooperative task (Gromann et al., 2013) and reduced activation in the ventral striatum, ventromedial prefrontal cortex, and anterior cingulate cortex in response to smiling face images (Lee et al., 2019). In the general population, social reward sensitivity is associated with positive affective experiences during pleasant social interactions (Laurenceau et al., 2010). In contrast, people with schizophrenia demonstrate diminished consummatory pleasure in laboratory-based social learning tasks (Campellone and Kring, 2018; Campellone et al., 2018). However, following social role-play (Aghevli et al., 2003) and when connecting with others in daily life (Mote and Fulford, 2020), people with and without schizophrenia do not differ in reported positive affect. Social reward sensitivity could help clarify these mixed findings. Aligned with Strauss et al.’s (2017) assertion that people with schizophrenia demonstrate a reduced positivity offset when motivational significance is low, as described previously, the social incentives used in laboratory-based tasks may be undetectable to individuals who have a diminished social reward sensitivity.

A defining feature of social reward sensitivity is the role of incentives for social connection. Empirical evidence suggests that those more sensitive to social reward are more likely to adopt goals and definitions of relationship quality that emphasize social incentives (Elliot et al., 2006; Gable and Poore, 2008). Adopting social approach-oriented goals, such as spending more quality time with family, is associated with less loneliness and more positive attitudes toward relationships (Gable, 2006). People with a higher social reward sensitivity prioritize the presence of relationship growth over security when defining relationship success (Gable and Poore, 2008; Cortes et al., 2018). Though recent work has identified that people with schizophrenia are more motivated by the affiliative aspects of social connection than by instrumental benefits or a sense of obligation (Weittenhiller et al., 2021), it is unclear to what degree this affiliative motivation stems from a desire to gain social incentives (e.g., companionship, support), rather than a desire to avoid social punishments (e.g., loneliness, rejection). It is possible that people with schizophrenia with social motivation difficulties undervalue social incentives. Future work that explores how people with schizophrenia evaluate social connection, frame social goals, and define relationship success, is thus warranted.

Social reward sensitivity has implications for social reward learning. After experiences of social acceptance, people with heightened social reward sensitivity are more likely to attribute positive social outcomes to internal, stable, and global causes (Schoch et al., 2015). These attributions, in turn, predict greater anticipation of future positive social experiences (Nikitin et al., 2019). Though little is known about how people with schizophrenia make sense of social acceptance (or rejection) experiences, studies suggest they anticipate less pleasure for future positive social interactions (Engel et al., 2016; Campellone and Kring, 2018); although we note that people with schizophrenia still learn from positive social interactions and anticipate more pleasure for subsequent interactions with the same people (Campellone and Kring, 2018). In the general population, individuals with higher social reward sensitivity exhibit a positive social memory bias (Strachman and Gable, 2006), and have greater regional gray matter volume in prospection and valuation brain areas, including the ventromedial prefrontal cortex (Crawford et al., 2020). Consequently, social prospection and memory abilities may play a role in social reward sensitivity and social pleasure expectancies.

A particular challenge for people with schizophrenia is the conversion of social interest into goal-directed behavior. Schizophrenia is associated with less willingness to take social risks (Kéri et al., 2009) or expend effort to increase the likelihood of positive social interactions (Campellone et al., 2018), compared to people without the illness. People with schizophrenia report less willingness to engage in approach behaviors toward socially rewarding stimuli (Radke et al., 2015) or other people (de la Asuncion et al., 2015). And those who exhibit more social withdrawal may be particularly prone to effort expenditure deficits (Fulford et al., 2018). In non-clinical undergraduate samples, social reward sensitivity corresponds with increased engagement in approach behavior (e.g., initiating new relationships; Nikitin et al., 2019) and more frequent positive social interactions in daily life (Gable, 2006). It follows that people with schizophrenia with a diminished sensitivity to social reward may be less likely to expend social effort or engage in social approach behaviors. Fortunately, research on the treatment of social anxiety disorder suggests that interventions that target the interpersonal beliefs and behaviors associated with diminished sensitivity to social reward can successfully increase social approach behaviors (Plasencia et al., 2016; Alden et al., 2018).

### Other Considerations When Studying Psychological Attributes in Relation to Motivation and Pleasure Deficits in Schizophrenia

#### Adversity, Stigma, and Stress

Although beyond the focus of this review, it is important to acknowledge the impact of early life adversity, psychosocial stress, and stigma on the formation of belief systems and cognitive schemas (Grant and Beck, 2009). This is highly relevant to schizophrenia, a disorder associated with high rates of childhood adversity (Matheson et al., 2013), which can impact cognitive development (Wells et al., 2020), and correspond with reward processing alterations throughout the lifespan (Dillon et al., 2009; Birn et al., 2017; Hanson et al., 2017; Herzberg and Gunnar, 2020). Barriers to social engagement, such as internalized stigma, rejection, and limited finances, are more common among people with schizophrenia (Weittenhiller et al., 2021). Diminished social reward sensitivity in schizophrenia may stem, in part, from an accumulation of negative social interactions. People with schizophrenia report more social stress in their daily lives (Mote and Fulford, 2020), and are more likely to experience stigma related to their condition than those with other serious illnesses, such as depression, cancer, or AIDS (Corrigan et al., 2000; Rossler, 2016). Stigma may play a role in the development and maintenance of low pleasure, success, and social acceptance expectancies (Rector et al., 2005); internalized stigma of mental illness is associated with worse defeatist performance beliefs and lower expectations for success in people with schizophrenia (Park et al., 2013). People with psychosis are also twice as likely as unaffected comparison participants to report bullying victimization (Trotta et al., 2013). CHR youth who experience bullying report more severe asocial and defeatist performance beliefs (Braun et al., 2021). Alternatively, adaptive beliefs, like those related to self-efficacy, are associated with resilience to adversity (Schwarzer and Warner, 2013), including after trauma (Luszczynska et al., 2009).

#### Positive Symptomology

Positive symptoms of schizophrenia, such as paranoia or suspiciousness, may also impact motivation, particularly in a social context. For instance, during a reversal learning task with social interactions as reward, placing more trust in a social partner correlated with better real-world social functioning, greater motivation, and less suspiciousness in people with schizophrenia (Campellone et al., 2016a). People with schizophrenia who experience paranoia may tend toward active social avoidance, rather than passive social withdrawal, which may further preclude social interaction (Hansen et al., 2009; Brown et al., 2014). Thus, though positive symptoms were not a focus of this review, the full range of schizophrenia symptomatology holds relevance for understanding amotivation and low social drive in this population.

#### Future Research Directions

As a complement to questionnaires for specific traits or characteristics, researchers can broadly assess an individual’s personality structure, incorporating measures that capture the full range of a given psychological dimension, from adaptive to maladaptive (or pathological). This is important given that the areas most developed in the literature to date pertain to how dysfunctional attitudes relate to goal deficits in schizophrenia, while positive characteristics like optimism and mindfulness are less well-studied in schizophrenia. In addition to questionnaires and inventories, researchers can adopt experimental procedures for capturing individual variation in psychological dimensions. One example is how Lefebvre et al. (2017) identified optimistic participants empirically, based on responses during a reward learning task. This approach provides a translational parallel with animal studies, where trait-like tendencies are similarly inferred from behavior patterns (e.g., the “optimistic” rats during an operant condition task; Rygula et al., 2015). In such cases, assessments are not biased by the participant’s self-report. Psychological characteristics can also be measured via experimental inductions, such as those described in studies of reappraisal and rumination (e.g., Whitmer et al., 2012; Bartolomeo et al., 2020); although more work is needed to understand how these experimental inductions relate to self-report assessments (especially given evidence for weak relationships between self-report and behavioral measures intended to capture the same construct; Dang et al., 2020).

These same psychological processes can inform treatment applications. People with schizophrenia report significantly less use of reappraisal and mindfulness and an overreliance on rumination. These, along with other psychological dimensions reviewed, are all processes that are addressed in common psychotherapeutic interventions. Key research questions are whether individual differences in baseline use of these processes can guide treatment selection, whether these processes can be modified in schizophrenia via interventions, and whether modifying these processes actually leads to meaningful improvements for people with schizophrenia. For example, across multiple study populations, mindfulness training techniques produce small to moderate increases in dispositional mindfulness measures, and changes in dispositional mindfulness correlate with improvements in other treatment outcomes related to mental health and well-being (meta-analysis by Quaglia et al., 2016). Mindfulness-based interventions are increasingly popular for schizophrenia (Jansen et al., 2020), and a logical next question is whether individual variation in dispositional mindfulness can help identify who will benefit most from those treatments. Conversely, baseline rumination tendencies may inform treatment outcomes, following recent evidence that lower levels of pretreatment rumination correlated with better quality of life at the end of treatment in people with anxiety disorders seeking CBT (Bredemeier et al., 2020). As noted above, defeatist performance and asocial beliefs contribute to real-world functioning and negative symptoms of schizophrenia (Campellone et al., 2016b; Thomas et al., 2017), rendering them important therapeutic targets (Granholm et al., 2018). Future work is needed to understand the relative contribution of these various psychological dimensions and beliefs to amotivation and anhedonia in schizophrenia, and to determine which components are malleable with treatment.

## Conclusion

This review covered how individual variation in psychological beliefs, and approaches for managing those beliefs, informs the study of motivation and pleasure in schizophrenia. We considered psychological features at the level of the individual as well as within an external, social context. We started with broader dispositional traits, such as optimism/pessimism, followed by propensities toward more specific negative beliefs about the self, others, and the future, and lastly covered strategies for managing thoughts and beliefs. While the literature regarding optimism and schizophrenia is rather small, insights from the general population and those with depression suggest that optimism may relate to reward anticipation. An increasing number of studies are examining how negative beliefs correlate with lower effort expenditure in schizophrenia, with the most consistent findings for defeatist performance beliefs. Psychological strategies that mitigate negative cognitions, like reappraisal and mindfulness are associated with reward responsivity. Comparatively, rumination has been associated with abnormal reward learning, as observed in non-psychiatric and depression samples. Finally, with respect to intrapersonal characteristics, social reward sensitivity relates to how an individual values social connection, how they learn from social experiences, and their willingness to expend effort for social rewards (although some of these relationships are yet to be tested in schizophrenia, directly).

There is a sizeable literature on the specific cognitive and reward processing features that underlie amotivation and anhedonia. What we propose here is that incorporating assessments of an individual’s beliefs about themselves, others, and the future may add to our understanding of motivation and pleasure functions in schizophrenia. Whether these psychological dimensions simply complement or interact with other cognitive and reward processing mechanisms is largely unknown. A more comprehensive study of these features, and their potential interactions, is needed to understand the relative contributions of these factors to motivation and pleasure deficits.

Amotivation and anhedonia remain unmet therapeutic needs (Galderisi et al., 2018) with profound impacts on real-world functional outcomes (Fervaha et al., 2014), even relative to neurocognitive deficits (Milev et al., 2005) and positive symptoms (Fenton and McGlashan, 1991; Rabinowitz et al., 2012). For several of the domains we covered, the relevant schizophrenia literature is small; consequently, these domains represent novel research directions warranting future study. While this review focused on schizophrenia, motivation processes have clear transdiagnostic relevance (Barch et al., 2016). Some of the psychological characteristics we cover are well-described in the clinical psychotherapeutic literature (like defeatist beliefs, mindfulness, and rumination), while others are prominent in personality research (like optimism). We underscore this opportunity to bridge these literatures with experimental clinical neuroscience research on amotivation and anhedonia to facilitate novel therapeutic development.

## Author Contributions

SA, LW, and SF substantially contributed to conception and writing the review, with SF providing oversight as the senior and corresponding author. CB conducted literature searches and edited the manuscript. JM, DM, and JF critically reviewed the manuscript. All authors read and approved the submitted version.

## Conflict of Interest

DM consults for Boehringer Ingelheim International, Cadent Therapeutics, Syndesi Therapeutics, Recognify Life Sciences, and Gilgamesh Pharmaceuticals. The remaining authors declare that the research was conducted in the absence of any commercial or financial relationships that could be construed as a potential conflict of interest.

## Publisher’s Note

All claims expressed in this article are solely those of the authors and do not necessarily represent those of their affiliated organizations, or those of the publisher, the editors and the reviewers. Any product that may be evaluated in this article, or claim that may be made by its manufacturer, is not guaranteed or endorsed by the publisher.
